# Benign Paroxysmal Positional Vertigo Risk Factors Unique to Perimenopausal Women

**DOI:** 10.3389/fneur.2020.589605

**Published:** 2020-10-16

**Authors:** Seong-Hae Jeong

**Affiliations:** Department of Neurology, Chungnam National University Hospital, Chungnam National University School of Medicine, Daejeon, South Korea

**Keywords:** vertigo, estrogen, women, otoconia, perimenopause

## Abstract

Many investigations have found common occurrences of benign paroxysmal positional vertigo (BPPV) in women, and clinical experience has shown that BPPV can develop due to increased hormonal fluctuations, especially during menopause. Therefore, knowledge about neurochemicals and their involvement with BPPV is imperative for the management of neurological issues in women. This review will discuss appropriate gender-based considerations of BPPV based on experimental and clinical evidence. The studies describe 2 lines of evidence regarding the association of perimenopause in women and the development of BPPV: (1) experimental evidence: the existence of estrogen receptors in the inner ear, otoconial malformations in osteopenic/osteoporotic rats, changes in otoconin 90 caused by hormone replacement therapy, and impaired calcium absorption following estrogen deprivation corrected by estrogen replacement therapy and (2) clinical evidence: epidemiological aspects, osteoporosis and estrogen deficiency. Future studies are necessary to validate the effects of hormonal replacement therapy and phytoestrogen in women with recurrent BPPV.

## Introduction

Known as the most common cause of recurrent vertigo (1), benign paroxysmal positional vertigo (BPPV) is an important health problem affecting more than 420 million adults worldwide, based on an international consensus that BPPV has a lifetime prevalence of 10% (2). BPPV increases with age, especially during menopause, in a ratio of 2-3.2:1 for women and men aged 40–60 years (3–8). In particular, hormonal changes, external estrogens, and pregnancy exposure are only experienced by women. Further understanding of possible contributors to the predominance of BPPV in women could make a significant contribution to our understanding of the causes of BPPV and may provide new methods for prevention. Therefore, the present study will review the current state of knowledge on BPPV risk factors specific to women.

## Perimenopause Period

There are three stages of perimenopause in the executive summary of the Stages of Reproductive Aging Workshop: “early menopausal transition (early perimenopause), characterized by irregularities in the menstrual cycle, late menopausal transition (late perimenopause), characterized by an interval of more than 60 days of amenorrhea in the previous 12 months; and early postmenopause, which is the first year following the last menstrual period (9).” Physiologically, estrogen declines sharply during this period, which occurs over several years and is characterized by marked fluctuations in sex hormone levels. The fluctuations during this period are more severe than those during the menstrual cycle (10). Regarding personal aspects, menopause occurs during the time in life when women are actively involved in raising a family and/or full-time work, during which time women may also be responsible for caring for elderly parents. Most menopausal women experience uncomfortable symptoms, and menopause is related to an increased risk for metabolic syndrome, in addition to obesity and osteoporosis (11). In the field of gynecology, menopausal symptoms are assessed using the “Kupperman rating scale” (Table 1) (12–14). The inclusion of vertigo items in one menopausal symptom index suggests that vertigo is also a frequent complaint in gynecology clinics. Although the nature of vertigo has not been precisely elucidated, postmenopausal hormone replacement therapy (HRT) was superior to placebo when assessed by the “Kupperman scale including the item addressing vertigo (15).” Additionally, many neurotology studies have shown an increased incidence of BPPV in women, and experience with older people suggests that hormonal fluctuations, particularly during menopause, may increase, resulting in the development of BPPV (3–8). Cooperative management between neurotologists and gynecologists could be helpful in the management of dizziness in women who experience it.

**Table 1:** The Kupperman scale*.

**Items**		**Factor**	**Severity**	Numerical Conversion (Factor * Severity)
1	Hot flashes	4		
2	Sweating	2		
3	Paresthesia	2		
4	Insomnia	2		
5	Nervousness	2		
6	Melancholia	1		
7	Vertigo	1		
8	Fatigue	1		
9	Myalgia	1		
10	Headache	1		
11	Palpitation	1		
12	Vaginal dryness	1		
Menopausal index (sum of each numerical conversion)				(0–57)

Severity: O—None = 0, S—Slight = 1, M—Moderate = 2, +–Marked = 3 *The Kupperman scale described by Morrie M. Gelfand in 2002.

## Experimental Evidence

### Anatomical Differences

Differences in the peripheral vestibular system between men and women have been reported. To our knowledge, no reports have described the direct comparison of otoconial morphology between women and men. However, in the anteroposterior dimension, the width of the peripheral vestibular system is significantly smaller in women (16), which could suggest a difference in otoconial morphology, such as differences in the bones according to sex. Further validation studies are needed.

### Hormone Changes

Physiologically, testosterone levels are more stable than estrogen levels throughout a woman's life and are relatively constant at ages 30–70 (17). Estrogen decreases steeply during the climacteric period. The female sex hormone estrogen 17β-estradiol (E2) is well-known to perform many tasks and contributes to various roles, such as reproductive organ differentiation and function, memory processes and bone metabolism (18, 19). Clinically, E2 and progesterone levels are clearly reduced in women with BPPV (20, 21); however, no difference in testosterone was found between the postmenopausal and control groups (21). Similar to other sex steroids, estrogen acts on target cells by attaching to nuclear hormone receptors such as estrogen receptor (ER)α and β (22). Thus, the ER level is an important determinant that influences estrogen signaling to the cell.

### Estrogen Receptors in the Inner Ear

Estrogen functions have been suggested to influence hearing and vestibular function (23, 24). In addition, the expression of ERα and β decreases with increasing age (25). A previous double-staining study found that in the inner ear, ERα and β were generally co-expressed (25). However, ERβ predominates in type II spiral ganglion neurons or inner ear strial marginal cells (24, 26–28). These findings support the role of estrogen in the inner ear.

### TRPV6 and Estrogen

BPPV is described as the process of otoconia debris removal from the otoconial membrane (29). Because otoconia are calcium carbonate crystals, initial crystal formation in the proteinaceous core needs a local increase in Ca^2+^ and carbonate (${\text{CO}}_{3}^{-}$) concentrations (30). Importantly, low Ca^2+^ levels must be maintained in the endolymph of vestibular organs to prevent unnecessary mineralization (31). Several Ca^2+^ channel structures primarily act on the temporospatial control of Ca^2+^ concentrations in the endolymph to preserve a circumstance suitable for stable Ca^2+^ equilibrium. Previous reports indicate that the epithelial Ca^2+^ channels TRPV5 and TRPV6 manifest in the semicircular canal, which are important Ca^2+^-binding proteins for the maintenance of low Ca^2+^ concentrations in the endolymph. Moreover, ERα has been demonstrated to tightly regulate uterine TRPV6 transcription. An experimental study also showed that E2 regulates TRPV6 through an ERα-dependent pathway (32). In a previous study, decreased ERα levels in aged animals could induce a decrease in TRPV6, resulting in otoconial malformation and a decrease in the number of otoconia (25). Therefore, a sharp decrease in estrogen during menopause, especially a decrease in ERα, may impair otoconial metabolism and lead to a higher prevalence of BPPV (25).

### Estrogen and Otoconia

Estrogen may also play an important role in otoconial metabolism. Bilateral ovariectomized osteopenic/osteoporotic rats had larger otoconia with a reduced density compared to controls (33).

### Estrogen and Otoconin 90

An animal study conducted by Yang et al. demonstrated that bilateral ovariectomy in rats receiving female HRT reversed the reduction in the level of otoconin 90, the main protein that preserves the normal morphology and growth of otoconia (20, 34).

### Estrogen and Vitamin D

Recent reports have shown that reduced serum levels of vitamin D are associated with the occurrence of BPPV (4, 35–40), and supplementation of vitamin D with/without calcium reduces recurrent events in BPPV patients (41–44). Estrogen treatment prevents the loss of intestinal Ca^2+^ absorption and bone density caused by ovariectomy in the premenopausal period (45). Experimental studies have shown that impaired Ca^2+^ absorption following estrogen deprivation is caused by a decreased response to 1,25 dihydroxycholecalciferol, the main regulator of intestinal absorption, and that estrogen treatment, rather than short-term replacement with 1,25 dihydroxycholecalciferol, can amend this abnormality. Therefore, combined hormone replacement and vitamin D management could be more effective for the prevention of further attacks of vertigo in perimenopausal women with BPPV. However, further validation studies are needed (45).

## Clinical Evidence

### Susceptibility of Women to BPPV

Several factors increase the susceptibility to BPPV, including older age, head and neck trauma, inactivity, and other ear problems or surgery. Many studies have shown a common occurrence in women, and clinical experience with older people has shown that BPPV can develop due to increased hormonal fluctuations, especially during menopause (3–8).

### BPPV and Oral Contraceptives

A previous study reported that recurrent BPPV was related to oral contraceptives (46). It has been postulated that oral contraceptives may induce disturbances in the water and electrolyte balance, variances in endolymph pH and abnormalities in carbohydrate or lipid metabolism, which may cause otoconial degeneration and subsequent otoconial detachment and BPPV (46).

### BPPV in Pregnancy

Although the link between BPPV and pregnancy is still unclear, some pregnant women have been first diagnosed with BPPV during pregnancy (47).

### Osteoporosis

A characteristic feature of osteoporosis is a decrease in bone mass due to an imbalance between bone resorption and formation. Loss of reproductive function and aging are the two most significant factors developing this condition. After ~30–40 years, both women and men experience 0.3–0.5% bone loss per year (48, 49). After menopause, the rate of bone loss can increase 10 fold. In Western countries, one-third of women with menopause experience osteoporosis (50). There are two types of osteoporosis: primary osteoporosis and secondary osteoporosis (51). Type I primary osteoporosis occurs during the postmenopausal period when estrogen output and the bone formation rate are reduced and bone loss is accelerated (51). Type II primary osteoporosis is a senile process with a reduction in the synthesis of active vitamin D in the elderly, which reduces gastrointestinal absorption of Ca^2+^, bone cell activity and bone development. Secondary osteoporosis is caused by specific conditions and drugs and occurs in all age groups irrespective of sex. Fractures and injuries due to osteoporosis could harm a person's life and incapacitate their ability to live independently. Therefore, bone fractures related to decreased bone density can increase mortality (51). In addition, decreased bone mineral density is related to the occurrence/recurrence of BPPV. Patients with BPPV had lower bone density among both women and men compared to controls (52). Although both men and women may develop osteoporosis, women are more susceptible to osteoporosis than men due to their smaller size with lower bone mass and decreased estrogen secretion during menopause (53, 54). In a previous study on bone mineral density and BPPV, women over the age of 45 in the relapse group had lower T scores than those in the *de novo* group (52). To examine osteoporosis in menopausal women, ovariectomized rats have been investigated in several studies (55, 56). Estrogen spares the human skeleton from bone loss by slowing down the process of bone remodeling and maintaining an equilibrium between bone formation and absorption (57, 58). In women undergoing rapid bone loss after menopause (59, 60), bone resorption and urinary Ca^2+^ excretion are increased, and these changes are reversed by estrogen replacement therapy (61, 62). Considering that estrogen loss is a causal factor for bone loss during perimenopause, a specific ER modulator could be used to treat postmenopausal BPPV patients.

### Hormone Replacement Therapy

HRT to combat estrogen depletion in menopause has also been successfully used to treat vasomotor symptoms and is assumed to be neuroprotective compared to women not using HRT. Although the nature of vertigo has not been precisely elucidated, women using postmenopausal HRT had better scores than the placebo group on the “Kupperman scale” (sweating, hot flashes, myalgia, and vertigo) (15). The hormonal fluctuation of ovarian neurosteroids might trigger the occurrence/recurrence of BPPV during the perimenopausal period. In the Taiwanese population, a study revealed that the incidence of BPPV was significantly lower in patients taking estrogen for menopausal syndrome in two age groups (ages 45–65 and ages 65 and over) (6). These findings support the efficacy of estrogen supplementation to decrease the occurrence of BPPV in women with menopause. The possible mechanisms include complete, more reliable estrogen blood levels that induce protective effects, estrogen effects on autophagy and possible epigenetic modulation (5, 6, 20, 63). However, chronic use of HRT increases the risk of breast cancer (64), stroke (64), and venous thrombosis. Therefore, many postmenopausal women rely on other non-steroidal estrogen mimetics or natural remedies to solve the problems associated with the symptoms of estrogen deficiency. Phytoestrogen could be an alternative to HRT. Phytoestrogens, including soy isoflavones, are non-steroidal, diphenolic substances that can bind to ERs and have activity similar to that of estrogen (65). To date, there have been no reports on the effectiveness of phytoestrogens on BPPV recurrence.

## Conclusion

The biochemical pathway is a key factor in some medical illnesses. Thus, investigation into the biochemical causes of neurotological problems could be guaranteed to produce results and can be cost effective. In particular, hormonal changes, external estrogens, and pregnancy exposure are only experienced by women. It is not fully understood why postmenopausal women show a higher prevalence of BPPV. However, in menopause, the rapid decrease in ERs, especially ERα, due to the sudden decrease in estrogen can lead to the disturbance of otoconial metabolism, which can increase the prevalence of BPPV. Additionally, HRT can reverse the low otoconin 90 levels and reduce the incidence of BPPV (Table 2). Adoption of a customized approach considering the neurochemical changes in perimenopause will be extremely helpful for the management of BPPV in women. Cooperative management among neurotologists, endocrinologists, and gynecologists is also important as a focus for neurotological disorders, including BPPV, in women.

**Table 2:** Evidence of the relationship of estrogen to the pathogenesis of benign paroxysmal positional vertigo.

	**References**	**Findings**
Experimental	(26)	In general, co-expression of ERα and β in the inner ear.
	(32)	TRPV6 is an important Ca^2+^ binding protein for maintenance of low [Ca^2+^] in the vestibular endolymph. Estradiol regulate TRPV6 via an ERα-dependent pathway.
	(33)	In rats by ovariectomy, the density of otoconia with larger size compared to the controls.
	(25)	Otoconial malformation and decreased the number of otoconia in older animals with ERα deficiency.
	(20)	The recovery of the otoconin 90, which preserves the normal morphology and growth of otoconia in bilateral ovariectomy in rats receiving female sex hormone replacement therapy
Clinical	(8)	Perimenopausal women with BPPV are the predominant patient type in dizziness clinics.
	(3)	
	(5)	
	(6)	
	(4)	
	(7)	
	(45)	Estrogen supplement, not short-term treatment with vitamin D, can repair calcium ion malabsorption occurring after estrogen deprivation.
	(46)	Recurrent BPPV is related to oral contraceptive treatment.
	(47)	Pregnant women with first attack of BPPV
	(6)	In the Taiwanese population, estrogen care for menopausal syndromes significantly lower incidence of BPPV.
	(20)	The lower level of estradiol in the postmenopausal women with idiopathic BPPV than those in the control subjects (*p* < 0.001).

*BPPV, benign paroxysmal positional vertigo*.

## Author Contributions

S-HJ acquired and analyzed the data, and drafted the manuscript.

## Conflict of Interest

The author declares that the research was conducted in the absence of any commercial or financial relationships that could be construed as a potential conflict of interest.
